# Operation in Appendicitis

**Published:** 1900-09-15

**Authors:** 


### Hospital Clinics and Medical Progress.

### OPERATION IN APPENDICITIS.

A strong tendency may be observed at present among certain advanced surgeons to lay down very definite rules as to the treatment of appendicitis, so definite, in fact, that if they should gain general acceptance, those who act in opposition to them may find it necessary to defend their advice and give reason for their "conservatism." Those who recognise how ready people outside the medical profession are to pick up smatterings, and to impute blame whenever a line of treatment which has become popularised is not adopted—as, for example, we have seen in recent years in regard to the non-use of the Röntgen rays or of antitoxin, or of certain antiseptic methods—must feel that the definite pronouncement that all cases of appendicitis should be operated on is one the acceptance of which, besides being a grave matter in itself, might lead to very embarrassing consequences. If we put on one side for a moment the acute and serious cases which obviously call for surgical consideration, and regard those alone in which many still think it justifiable to have recourse to medical treatment, we see that we often have no means of deciding, with anything approaching certainty, whether the individual case is one of the lucky ones which will escape further trouble, or belongs to the unfortunate group, and, after suffering many things from many physicians, will ultimately come under the surgeon. This was rendered abundantly clear in a discussion which took place early this year at the Medical Society of London; for although the remarks made by Dr. Allchin and Dr. Cayley tended to show that, in a considerable proportion of instances, attacks of appendicitis are recovered from, and are not followed by recurrence, it must be confessed that the discussion was anything but illuminating as to the means of deciding into which category a given case is to fall. The remarks made on the same occasion by Mr. Lockwood and others went strongly to suggest that even in some of the cases which might appear the most innocent such an amount of septic mischief might be lying *perdu*, perhaps merely dormant, as to point strongly to operation as the safest way out of the difficulty. In America the latter view has taken a strong hold, and it is there commonly maintained that, in view of the doubt which must hang over the future of every case, the safer course is to operate while the operation can be done under good conditions, rather than leave the patient to run the risk of having to face the ordeal when he may be far from experienced surgical help or may be amid less favourable surroundings. There is a great deal to be said for the position taken up by those who advocate this view, and exactly in proportion as the risks of operation are lessened so is the force of this line of argument likely to prevail. Lately, however, a still more advanced position has been maintained. It is now held that however free from recurrence patients may remain after an attack of appendicitis they are never cured unless the appendix be removed, and that, in fact, they always remain in a condition which predisposes to subsequent attack. It is held that non-operative cure is impossible, and that to leave a patient with a once-diseased appendix in his peritoneum is to leave him in a constant state of peril. The position is well stated in a recent summary of the matter from the advanced point of view in the *New York Medical Record*, where it is said that "those who are most radical in their views form a group which is receiving constant accessions of men who are generally inclined to be aggressive in their advocacy of what they think is right, and are pretty successful in supporting themselves with logic and statistical material," and that the position taken up by these gentlemen involves acceptance of the following statements: That appendicitis is always a grave and important disease; that it is never cured spontaneously; that the attacks are very apt to increase in severity; that the course of an individual attack involves several important elements of uncertainty; that there is no real non-operative treatment of the disease, though the symptoms can be mitigated and temporarily removed; and that operative treatment in competent hands, undertaken at the proper time, gives an exceedingly small mortality figure, which is unquestionably becoming smaller, and is unapproached by any form of non-operative treatment. Now this position may to some seem exceedingly radical. But see the simplification of the problem which it offers. No one can question that in many cases it is the delay and the hesitation—due to doubt and to the hope that the attack may not be repeated—that is the cause of many of the serious complications and the consequent bad results which are met with in operations for appendicitis, and no one can help seeing that if it is really to be accepted as true that operation is the one thing needful in every case, it will far more frequently happen to the surgeon to be able to choose the time which is most suitable for operation than is the case at present. According to such a view the duties of the physician would end with the diagnosis of appendicitis, while the surgeon would no longer have to discuss the question, Whether to operate or not? but merely to decide the question, When? Nothing could be more simple. All, however, depends upon the "if," and upon the acceptance of the notion that an appendix once septic must be always septic, and that as long as it is in this condition it must be a cause of danger. The assertion of surgeons who, operating in simple cases and in the "interval," find the appendix stuffed full of pathogenic microbes, may be taken, and is taken by some, as pointing positively to the necessity of removing such an organ in the shortest and the simplest way. But there is another side even to that proposition. We must not lose sight of the fact, stated by physicians of experience, and admitted even by the most advanced surgeons, namely, that a very considerable proportion of cases do not recur; and it is only fair to hold that the more clearly surgeons prove that the appendices in these non-recurring cases contain septic organisms, the more should we doubt whether the presence of such organisms in an appendix is necessarily of serious import. Anyway, we are driven to appeal to experience rather than to bacteriology in deciding whether we ought to operate or not. Thus we come back to the question, What does experience teach?

At present the "radicals" have things much their own way. They can show how dangerous is delay, and how small is the mortality of operation in simple cases, and they boldly maintain that if every case were operated on there would be a considerable gain of life. Meanwhile, the "conservatives" can only say that a good many cases recover. It lies, then, with them to play the next move, and to bring the matter to a practical issue by showing how to tell in which cases non-recurrence may fairly be expected, and in which the operating surgeon must be asked to intervene. Of course there are clues, but we fear that there is at present nothing very positive to oppose to the extremely definite programme of the radicals; which is to be regretted, for this programme savours of routine, and a routine which involves operating on many cases which might get well without operation cannot be regarded as entirely satisfactory.

